# Identification and validation of a novel signature as a diagnostic and prognostic biomarker in colorectal cancer

**DOI:** 10.1186/s13062-022-00342-w

**Published:** 2022-11-02

**Authors:** Di Wang, Junye Liufu, Qiyuan Yang, Shengqun Dai, Jiaqi Wang, Biao Xie

**Affiliations:** 1 Department of Gastroenterology, People’s Hospital of Longhua, NO.38 Jinglong Construction Road, Longhua District, 518109 Shenzhen, P.R. China; 2 Department of Gastroenterology, Guangzhou First People’s Hospital, 511458 Guangzhou, P.R. China

**Keywords:** Colorectal cancer, Differentially expressed genes, Diagnostic model, Prognostic model

## Abstract

**Background:**

Colorectal cancer (CRC) is one of the most common malignant neoplasms worldwide. Although marker genes associated with CRC have been identified previously, only a few have fulfilled the therapeutic demand. Therefore, based on differentially expressed genes (DEGs), this study aimed to establish a promising and valuable signature model to diagnose CRC and predict patient’s prognosis.

**Methods:**

The key genes were screened from DEGs to establish a multiscale embedded gene co-expression network, protein-protein interaction network, and survival analysis. A support vector machine (SVM) diagnostic model was constructed by a supervised classification algorithm. Univariate Cox analysis was performed to construct two prognostic signatures for overall survival and disease-free survival by Kaplan–Meier analysis, respectively. Independent clinical prognostic indicators were identified, followed by univariable and multivariable Cox analysis. GSEA was used to evaluate the gene enrichment analysis and CIBERSORT was used to estimate the immune cell infiltration. Finally, key genes were validated by qPCR and IHC.

**Results:**

In this study, four key genes (DKC1, FLNA, CSE1L and NSUN5) were screened. The SVM diagnostic model, consisting of 4-gene signature, showed a good performance for the diagnostic (AUC = 0.9956). Meanwhile, the four-gene signature was also used to construct a risk score prognostic model for disease-free survival (DFS) and overall survival (OS), and the results indicated that the prognostic model performed best in predicting the DFS and OS of CRC patients. The risk score was validated as an independent prognostic factor to exhibit the accurate survival prediction for OS according to the independent prognostic value. Furthermore, immune cell infiltration analysis demonstrated that the high-risk group had a higher proportion of macrophages M0, and T cells CD4 memory resting was significantly higher in the low-risk group than in the high-risk group. In addition, functional analysis indicated that WNT and other four cancer-related signaling pathways were the most significantly enriched pathways in the high-risk group. Finally, qRT-PCR and IHC results demonstrated that the high expression of DKC1, CSE1L and NSUN5, and the low expression of FLNA were risk factors of CRC patients with a poor prognosis.

**Conclusion:**

In this study, diagnosis and prognosis models were constructed based on the screened genes of DKC1, FLNA, CSE1L and NSUN5. The four-gene signature exhibited an excellent ability in CRC diagnosis and prognostic prediction. Our study supported and highlighted that the four-gene signature is conducive to better prognostic risk stratification and potential therapeutic targets for CRC patients.

**Supplementary Information:**

The online version contains supplementary material available at 10.1186/s13062-022-00342-w.

## Background

Colorectal cancer (CRC) is a common malignant tumor worldwide and also one of the leading causes of cancer-related mortality [1]. The mortality rate of CRC is second and third highest-ranking cancer in male and female patients, respectively, in the USA and fifth in China [2, 3]. The 5-year survival rate of CRC patients is exceed 90% when diagnosed at early stages. Due to the lack of adequate diagnostic methods, CRC is often diagnosed at an advanced stage, and the 5-year survival rate for CRC patients diagnosed with metastasis is low at approximately 12% [4]. At present, despite the significant improvements in diagnosis and treatment, the early diagnosis of CRC continues to be a global problem [5]. It is urgently needed that more effective diagnosis and prognostic evaluation systems to provide personalized medicine and improve outcome for CRC patients [6]. With the development of biology, biomarkers exhibited an increasingly important role in the early diagnosis, prognostication, survival, and clinical treatment monitoring of cancers. They have driven the development of personalized therapy and had a positive impact on patient outcomes [7]. Therefore, it is significant to identify and explore sensitive diagnostic and prognostic biomarkers for CRC.

With the advancement of gene chips and high-throughput second-generation sequencing technologies, the amount of publicly available high-throughput data is stored in global databases. Therefore, the combination of gene expression data with bioinformatics methods can be used to elucidate the expression of differentially expressed genes (DEGs) in the development and progression of CRC, as well as identify potential targets for the treatment of CRC. Recent studies have proposed DEGs as potential diagnostic and prognostic markers in CRC. Huang et al. identified hundreds of CRC-associated DEGs based on the Gene Expression Omnibus (GEO) and The Cancer Genome Atlas (TCGA) database. Thereinto, five could be used as diagnostic biomarkers for CRC patients [8]. Hou et al. identified a cluster of DEGs and DNA methylation aberrations in CRC. The results indicated that the combination of DEGs, DNA methylation aberrations, and tumor stages results in effective prognostic evaluation of patients with CRC [9]. Although some multigene-based prognostic signatures that can assess the prognostic risk of CRC had been established over the past 20 years, the robustness of most markers is less than expected and rarely could be used for CRC diagnosis, due to the inherent genetic heterogeneity of CRC [7, 10]. Therefore, finding effective and reliable signatures for the diagnosis and prognosis assessment of CRC patients is paramount and urgent.

In this study, four hub genes were identified from the DEGs by constructing a multiscale embedded gene co-expression network analysis (MEGENA), protein-protein interaction (PPI) network, and survival analysis. Then, to explore whether these key genes related to the diagnosis and prognosis of CRC, we constructed an SVM model and established a multi-gene signature for the prognosis of CRC patients by univariate and multivariable Cox regression analyses. This model adequately predicted the overall survival (OS) and disease-free survival (DFS) of CRC patients and provided a theoretical basis for the future study of CRC diagnosis and the underlying molecular mechanism.

## Results

### Identification of DEGs

**Fig. 1:**
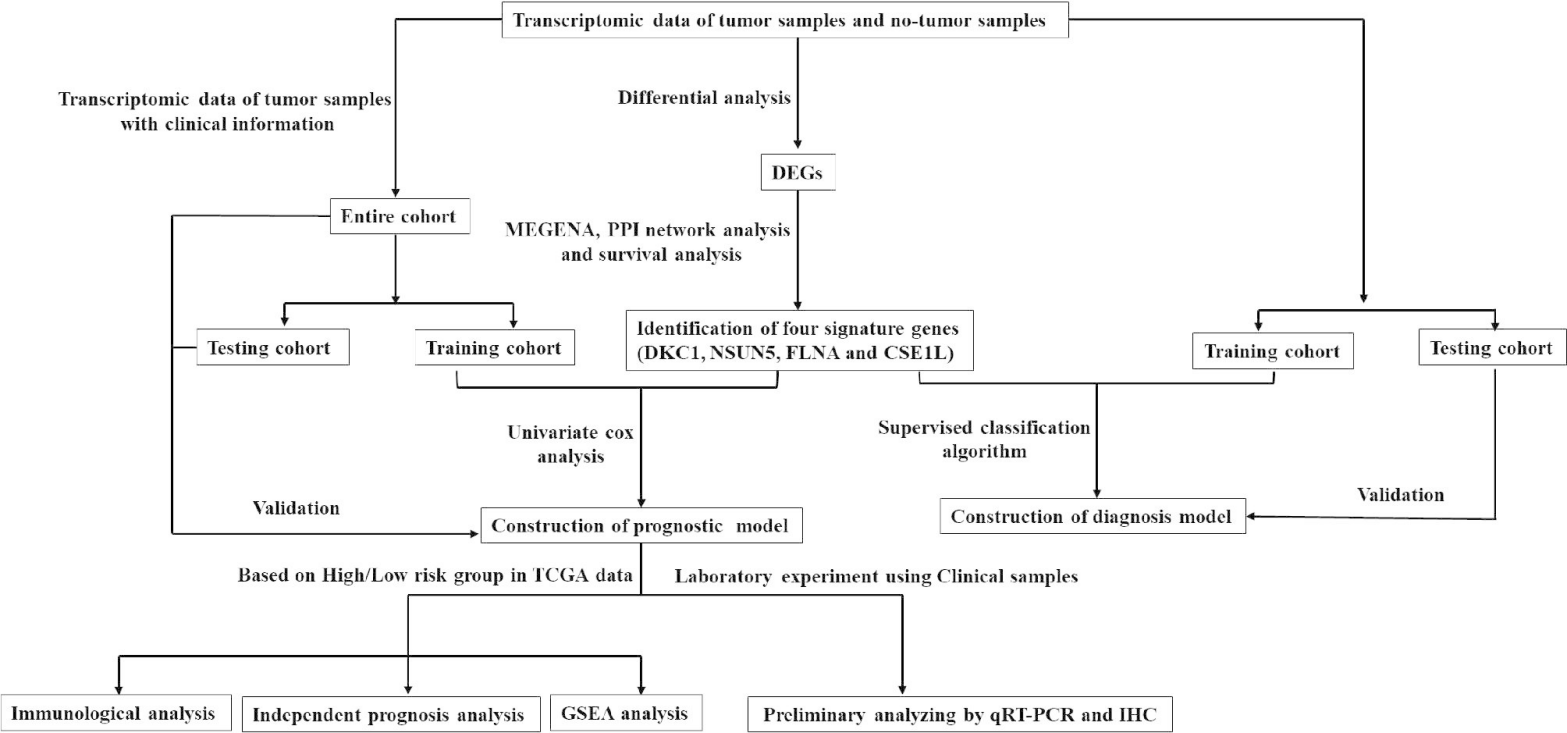
Flowchart of the study

**Fig. 2:**
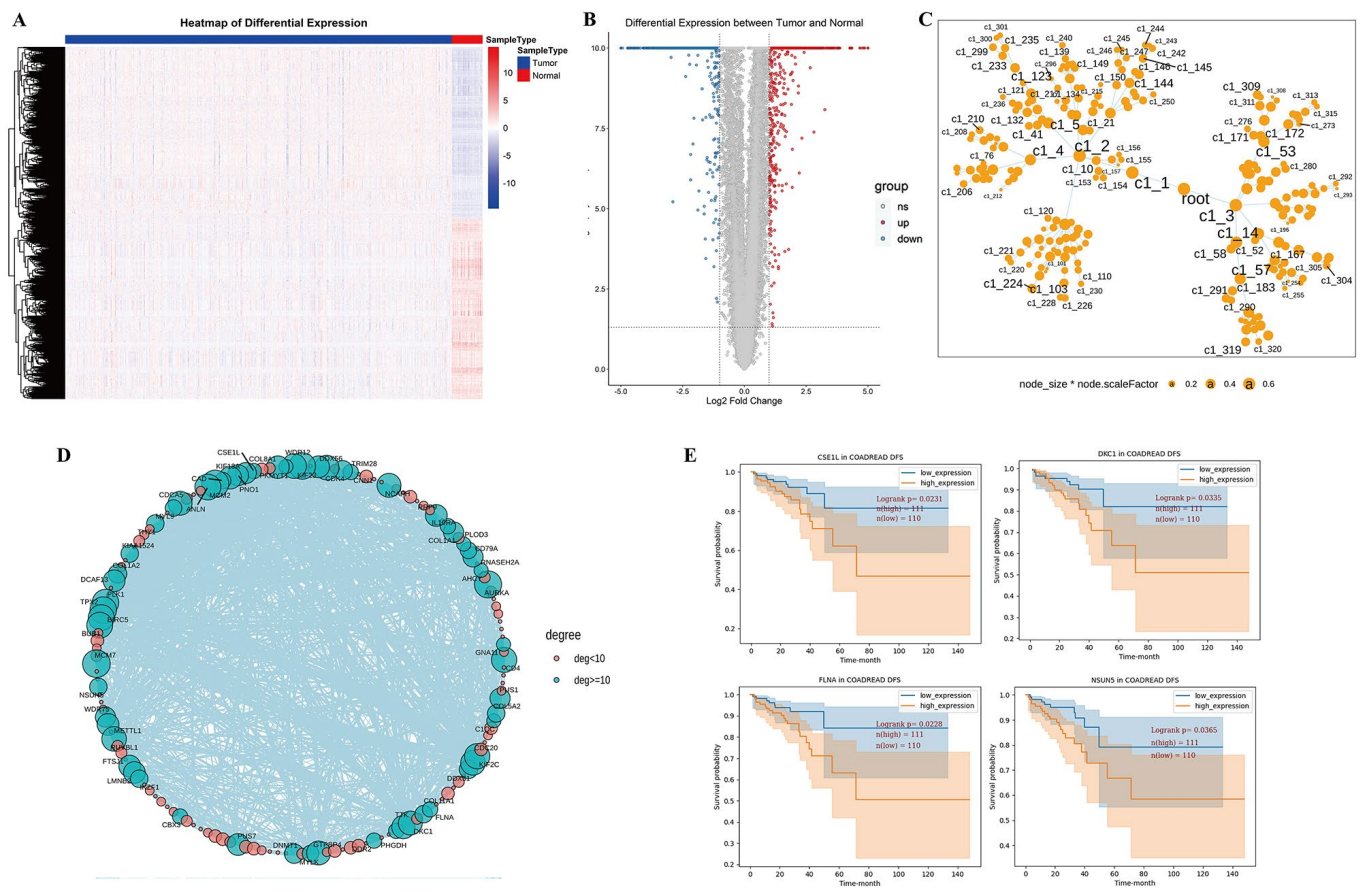
Identification of a four‑gene signature in CRC patients. (A) Heatmap of significant DEGs based on the expression level; (B) The volcano figure to show the upregulated and downregulated genes; (C) MEGENA among differentially expressed genes; (D) PPI network among node genes from the MEGENA; (E) Kaplan–Meier curves with the log-rank test were performed on disease free survival analysis of hub genes (DKC1, NSUN5, FLNA and CSE1L) from the PPI network

The current study is illustrated in the flow chart (Fig. 1). In order to determine the DEGs between the CRC tumors and adjacent tissues, p < 0.01 and |log2FC| > 2 were used as the threshold. The DEGs were displayed in the heatmap and volcano plot (Fig. 2 A and B). The results showed that compared with paracancerous tissue, 3409 genes were significantly upregulated and 3410 genes were significantly downregulated in CRC tissue.

### Identification of node genes

To identify the compact gene modules based on the 6819 DEGs, MEGENA method was used to construct a gene-gene causal network. The results showed that a total of 237 genes were cluster analyzed to identify the DEGs (Fig. 2 C). Finally, these gene networks identified 337 highly connected DEGs for downstream analyses.

### PPI network analysis of DEGs

The STRING platform was used to construct a PPI network of 337 DEGs (Fig. 2D). A total of 61 nodes were included in the network based on the interaction score criteria (degree > 10). A total of 61 genes (MCM7, AURKA, MCM2, KIF23, PLK1, TPX2, ANLN, BIRC5, BUB1, CD4, CDC20, CDK4, WDR12, RUVBL1, KIF2C, NCAPH, CDCA, GTPBP4, TTK, DKC1, PKMYT1, NOP58, RRP9, DDX56, DCAF13, METTL, FTSJ1, LMNB2, PUS7, KIF18A, PNO1, RNASEH2A, COL1A1, WDR75, PUS1, TRIM28, DNMT1, COL1A2, DDX31, THY1, KIAA1524, NSUN5, IKZF1, FLNA, COL5A2, CD79A, CAD, MYL9, MYLK, IL10RA, CNN1, CSE1L, CBX3, DDR2, PHGDH, COL11A1, GNA11, AHCYPLOD3, COL8A1 and C1QC) were selected as the hub genes.

### Survival analysis of hub genes

To further identify the key genes, we analyzed the correlation between hub gene expression and bioinformatics of CRC, and found that the DFS rate of high-expressing DKC1 was lower than that of the low-expressing molecule (p = 0.0335). Compared to the low-expressing NSUN5, the DFS rate of high-expressing NSUN5 was lower (p = 0.0365). Compared to the low-expressing FLNA, the DFS rate of the high-expression group of FLNA was lower (p = 0.0228). Meanwhile, the DFS rate of the high-expression of CSE1L was lower than the low-expressing CSE1L (p = 0.0231). No significant correlation was established between the expression of the other 57 hub genes and CRC prognosis (p > 0.05). These results identified DKC1, NSUN5, FLNA and CSE1L as the four key genes (Fig. 2E).

### Establishment and validation of the diagnostic model

**Fig. 3:**
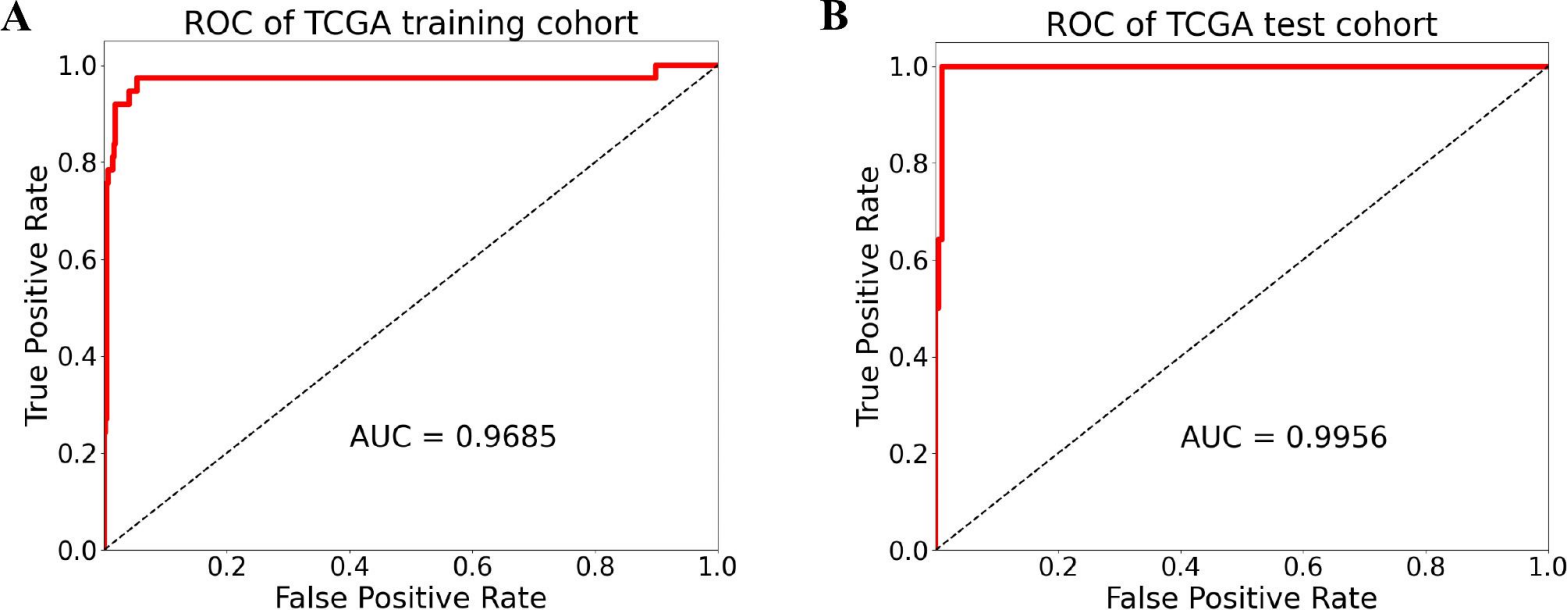
Establishment and validation of the diagnostic model of SVM. (A) ROC curve of SVM based on four genes as features based on training data. The x‑axis indicates the false positive rate, and the y‑axis indicates the true positive rate. The five‑fold cross‑validation is represented. The final fitted average is denoted by the black dotted line; (B) The ROC curve analysis for model efficacy based on testing data. The x‑axis represented the FPR, and the y‑axis represented the TPR. ROC: receiver operating characteristic, FPR: false-positive rate, TPR, true-positive rate

All four genes were statistically significant (p < 0.05) in the COADREAD dataset from TCGA after survival analysis. To construct and validate the four-gene diagnostic model, a total of 689 CRC samples in COADREAD datasets from TCGA were divided into the training (n = 482) and test sets (n = 207). A python 3.8 package scikit-learn was employed to construct the four-gene diagnostic model of SVM using the training set. The overall accuracy (fraction of correctly classified samples) of the binomial classifier for cross-validation on the training datasets was 0.9685 (Fig. 3 A), and the optimal parameter combination of the diagnostic model was determined (“C”: 23.2676, “gamma”: 0.4498). To further validate the current model, we evaluated the classifier on the test datasets; the area under curve (AUC) was 0.9956 (Fig. 3B). These results indicated that the constructed SVM-based classifier had high diagnostic values for the CRC patients.

### Establishment and validation of prognostic model

**Fig. 4:**
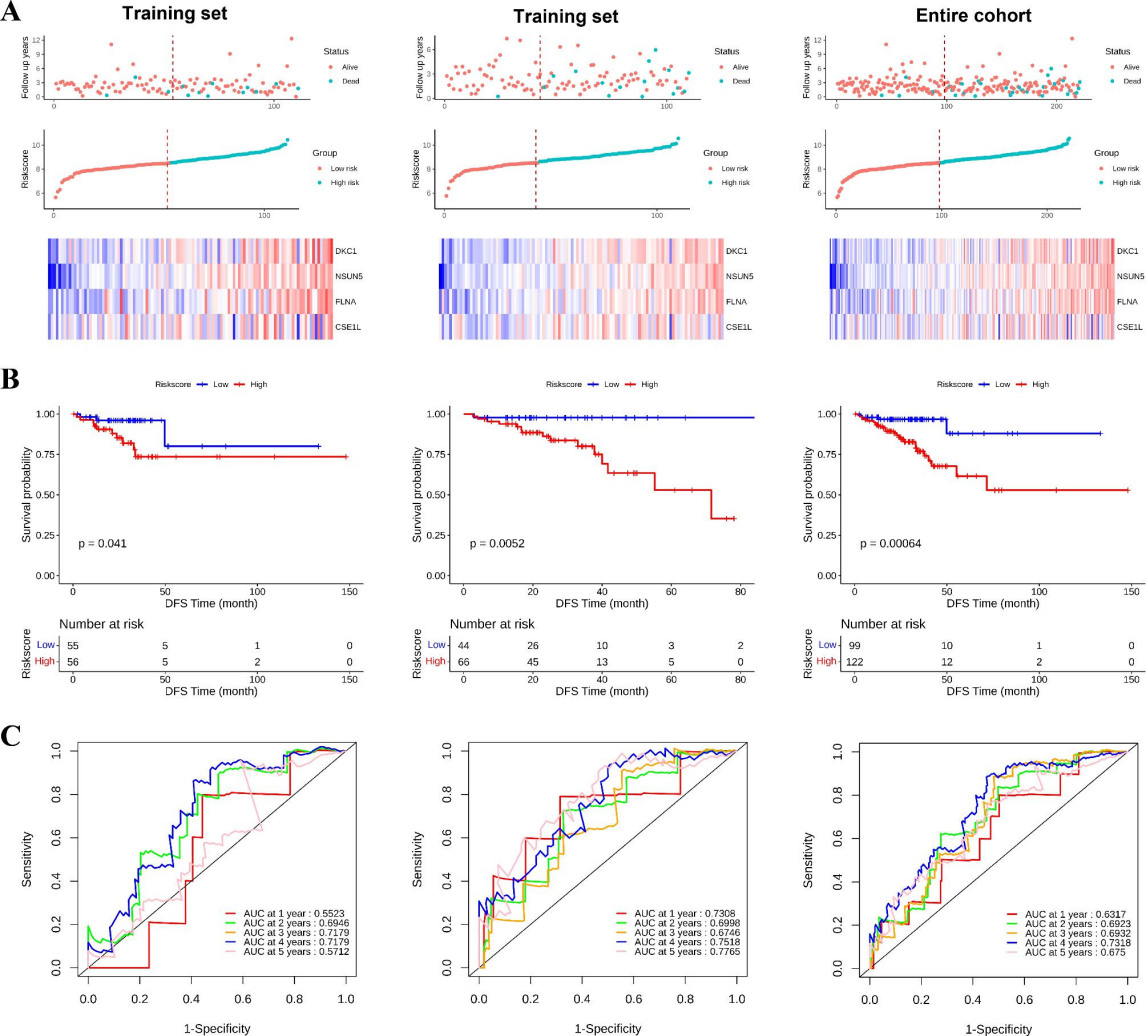
Establishment and validation of the prognostic model for DFS of patients in the training set, the test set, and the entire cohort. (A) The distribution of risk scores, gene expression levels, and patient relapse status; (B) Kaplan–Meier curves of DFS of the low-risk and high-risk groups; (C) ROC curve for the 1-, 2-, 3-, 4-, and 5-year survival prediction by the four-gene signature, respectively. The black dotted line represents the median risk score cutoff dividing the patients into low- and high- groups

A total of 221 CRC patients with the DFS data collected from the merged cohort of the TCGA and clinical information (DFS) were recruited, the detailed clinical features of ccRCC patients were listed in Supplementary material Table S1. Four genes were selected by survival analysis for constructing the prognostic model of DFS. The risk score formula consisting of the four DEGs was established for the prognostic model as follows: Risk score = (0.6374×DKC1)+(0.7798×NSUN5)+(0.2717×FLNA)+(-0.2354×CSE1L) (Table 1). Then, 221 CRC patients were divided into the training set (n = 111) and test set (n = 110). Next, we presented the risk score distribution curve and survival status of the training set (Fig. 4 A). The results showed that there were more deaths in the high-risk group, while most of the patients in the low-risk group stayed alive during the follow-up. The heatmap revealed the expression patterns of four DEGs between the two risk groups (Fig. 4 A). Based on the median prognostic risk score, the patients in the training set were divided into either the high-risk group (n = 56) or the low-risk group (n = 55). The K–M survival curve suggested that the patients of high prognostic risk group had poor DFS than the low risk patients (p < 0.05, Fig. 4B). The time-dependent ROC curve analysis was used to evaluate the predictive value of the signature. The AUCs of the training set at 1, 2, 3, 4, and 5 years were 0.5523, 0.6946, 0.7179, 0.7179, and 0.5712, respectively (Fig. 4 C). The formula mentioned above was used to calculate the risk scores, and the patients were also classified into high- and low-risk groups in the test and total sets based on the median value of 8.5343. The risk score distribution curve, survival status, and four genes expression heatmap of the test and total sets are shown in Fig. 4 A. Similarly, the predictive capability and clinical utility of the prognostic model was validated in both datasets. The results showed that the DFS of the high-risk group was significantly shorter than that of the low-risk group in both the test and totals set, according to the K–M survival analysis (p < 0.05, Fig. 4B), and the AUC for 1-, 2-, 3-, 4-, and 5-year DFS was 0.7308, 0.6998, 0.6746, 0.7518, and 0.7765 in the test set, respectively (Fig. 2E). In the total set, the AUCs for 1-, 2-, 3-, 4-, and 5-year DFS were 0.6317, 0.6923, 0.6932, 0.7318, and 0.675, respectively (Fig. 4 C). These results indicated the four-gene prognostic signature had the ability to predict DFS.

**Table 1:** Univariate regression analysis of four-signature genes for DFS

Symbol	Beta	Hazard ratio (95% CI)	Wald Test	p-value
DKC1	0.6374	1.8916(0.7266–4.9248)	1.3057	0.1917
NSUN5	0.7798	2.1811(0.8753–5.4346)	1.6741	0.0941
FLNA	0.2717	1.3122(0.9170–1.8777)	1.4860	0.1373
CSE1L	-0.2354	0.7902(0.3783–1.6507)	-0.6264	0.5311

**Fig. 5:**
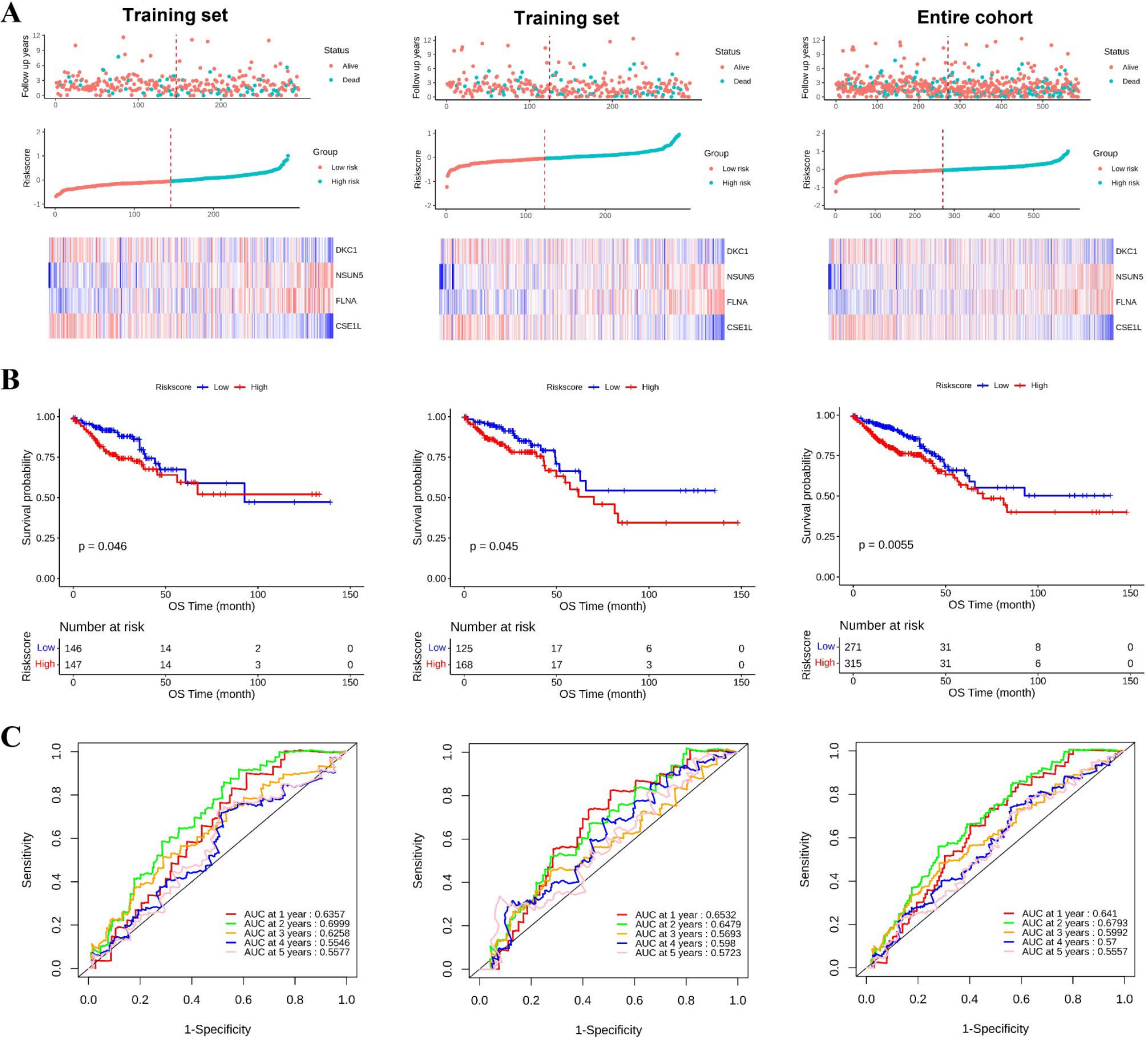
Establishment and validation of the prognostic model for OS of patients in the training set, the test set, and the entire cohort. (A) The distribution of risk scores, gene expression levels, and patient relapse status; (B) Kaplan–Meier curves of DFS of the low- and high-risk groups; (C) ROC curve for the 1-, 2-, 3-, 4-, and 5-year survival prediction by the four-gene signature, respectively. The black dotted line represents the median risk score cutoff dividing patients into low- and high- groups

Simultaneously, to determine the association between the gene expression signatures of these four genes and the CRC patient OS, the risk score of the four DEGs was calculated using univariate Cox regression analysis as follows: Risk score = (-0.1051×DKC1)+(0.3303×NSUN5)+(0.0960×FLNA)+(-0.2391×CSE1L) (Table 2). The detailed clinical features of ccRCC patients were listed in Supplementary material Table S1. A total of 587 patients with OS data were divided into the training set (n = 294) and test set (n = 293). Based on the median risk score of -0.0477, the training set (n = 294), the test set (n = 293), and the total set (n = 587) were divided into the high- and low-risk groups. The risk score distribution curve, survival status, and the four genes’ expression heatmap of the training, test, and total sets are shown in Fig. 5 A. The results showed that the patients of high prognostic risk group had a higher mortality than the patients of low prognostic risk group. The heatmap revealed the expression patterns of four DEGs between the two risk groups. Compared to the low-risk group, a significantly poor OS was observed in the high-risk group by the K–M survival curve analysis (p < 0.05, Fig. 5B). We also performed the time-dependent ROC curve analysis, and the AUCs for 1-, 2-, 3-, 4-, and 5-year DFS were 0.6532, 0.6479, 0.5693, 0.598, and 0.5723, respectively, in the test set (Fig. 5 C). In the total set, the AUCs for 1-, 2-, 3-, 4-, and 5-year DFS were 0.641, 0.6793, 0.5992, 0.57, and 0.5557, respectively (Fig. 5 C). These results indicated that the prognostic model performed best in predicting the OS risk of CRC patients.

**Table 2:** Univariate regression analysis of four-signature genes for OS

Symbol	Beta	Hazard ratio (95% CI)	Wald test	p-value
DKC1	-0.1051	0.9002(0.5513–1.4700)	-0.4202	0.6744
NSUN5	0.3303	1.3915(0.9472–2.0441)	1.6835	0.0923
FLNA	0.0960	1.1007 (0.9529–1.2714)	1.3046	0.1920
CSE1L	-0.2391	0.7873 (0.5377–1.1529)	-1.2286	0.2192

### Gene set enrichment analyses (GSEA) of the prognostic signature

**Fig. 6:**
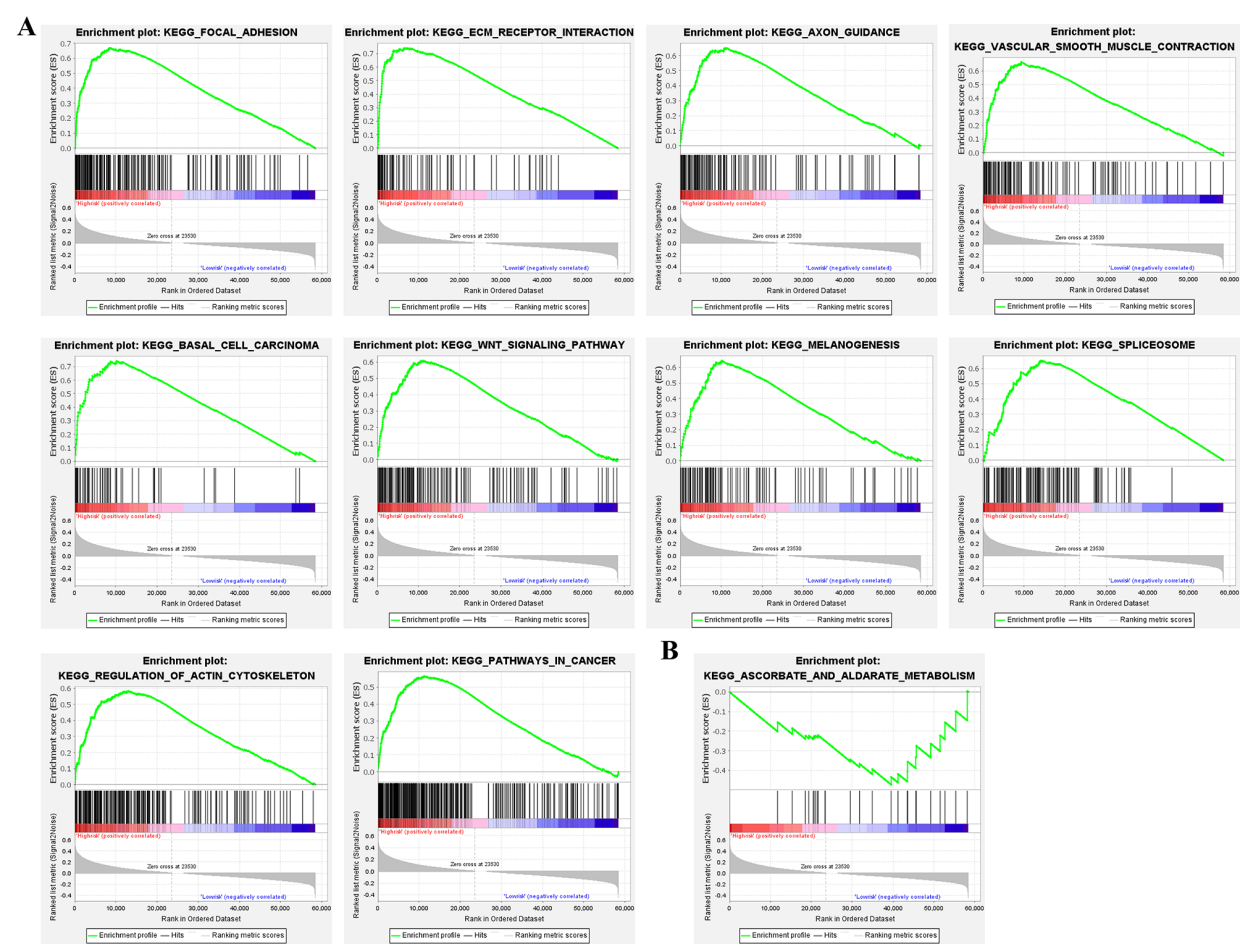
Functional enrichment analysis of genes correlated with signature genes in the high- and low-risk groups via GSEA. (A) Top 10 of KEGG enrichment analysis of signature genes in the high-risk group; (B) Only one in the low-risk group

We performed GSEA on the two risk groups to further explore the critical KEGG pathways. The results showed that 164 KEGG enriched pathways were active in the high-risk group, while 13 were active in the low-risk group. Furthermore, 101 statistically significant KEGG pathways (p < 0.05, FDR < 0.25) were screened, among which 100 were active in the high-risk group, while only one was active in the low-risk group. The top 10 pathways with the highest NES (normalized enrichment score) in the high-risk group and one pathway in the low-risk group (Table 3) were selected for visualization analysis (Fig. 6 A–B). The results showed that focal adhesion [11], ECM receptor interaction [12], AXON guidance [13], basal cell carcinoma [14], and WNT signaling pathway [15], which were onsidered to be associated with the poor prognosis of CRC, were the most significantly pathways enriched in the high-risk group.

**Table 3:** Ten KEGG pathways in the high-group and one in the low-group

Name	NES	p-val	FDR
High-risk group			
KEGG_FOCAL_ADHESION	2.9114	0	0
KEGG_ECM_RECEPTOR_INTERACTION	2.8482	0	0
KEGG_AXON_GUIDANCE	2.6994	0	0
KEGG_VASCULAR_SMOOTH_MUSCLE_CONTRACTION	2.6843	0	0
KEGG_BASAL_CELL_CARCINOMA	2.6754	0	0
KEGG_SPLICEOSOME	2.6402	0	0
KEGG_WNT_SIGNALING_PATHWAY	2.5723	0	0
KEGG_MELANOGENESIS	2.5571	0	0
KEGG_REGULATION_OF_ACTIN_CYTOSKELETON	2.5567	0	0
KEGG_PATHWAYS_IN_CANCER	2.5515	0	0
Low-risk group			
KEGG_ASCORBATE_AND_ALDARATE_METABOLISM	-1.5729	0.0143	0.172

### Independent prognosis analysis in the TCGA dataset

Independent prognosis analyses of clinical characteristics (age, gender, tumor stage and pathological TNM (tumor-node-metastasis)) and risk score determined the patient survival. Univariate and multivariate Cox regression analyses were performed on the clinical characteristics and risk scores of the overall samples from the TCGA dataset. As shown in Table 4, the univariate Cox regression analyses evaluated the prognostic value of the signature (high risk/low risk, p = 0.0022), N stage (N0/N1, p = 0.0328), and gender (female/male). However, the multivariate Cox regression analysis showed that risk score and gender were significantly related to prognosis for OS. Therefore, the results of univariate Cox and further multivariate Cox analyses validated that risk score was an independent prognostic factor for CRC. These results suggested that the prognostic signature was an accurate model for predicting prognosis of patients with CRC.

**Table 4:** Analysis of clinical characteristics and risk-score for the effectiveness of the prognostic signature

Variables		Univariate analysis				Multivariable analysis			
		HR	95% CI of HR	p	HR		95% CI of HR	p	
Risk score									
Low-risk									
High-risk	5.1908		1.8055–14.924	0.0022	4.838		1.6668–14.044		0.0037
Age									
< 60									
≥ 60	0.8458		0.3945–1.8134	0.667					
Stage									
I-II									
III-IV	1.1476		0.526–2.5039	0.7294					
N stage									
N0									
N1	2.662		1.0831–6.5424	0.0328	2.09		0.8423–5.184		0.1119
T stage									
T1-T2									
T3-T4	2.1384		0.7408–6.1725	0.1599					
gender									
Female									
Male	2.2676		1.0336–4.9749	0.0411	2.378		1.0811–5.232		0.0313

### Analysis of immune cell infiltration of the prognostic signature

**Fig. 7:**
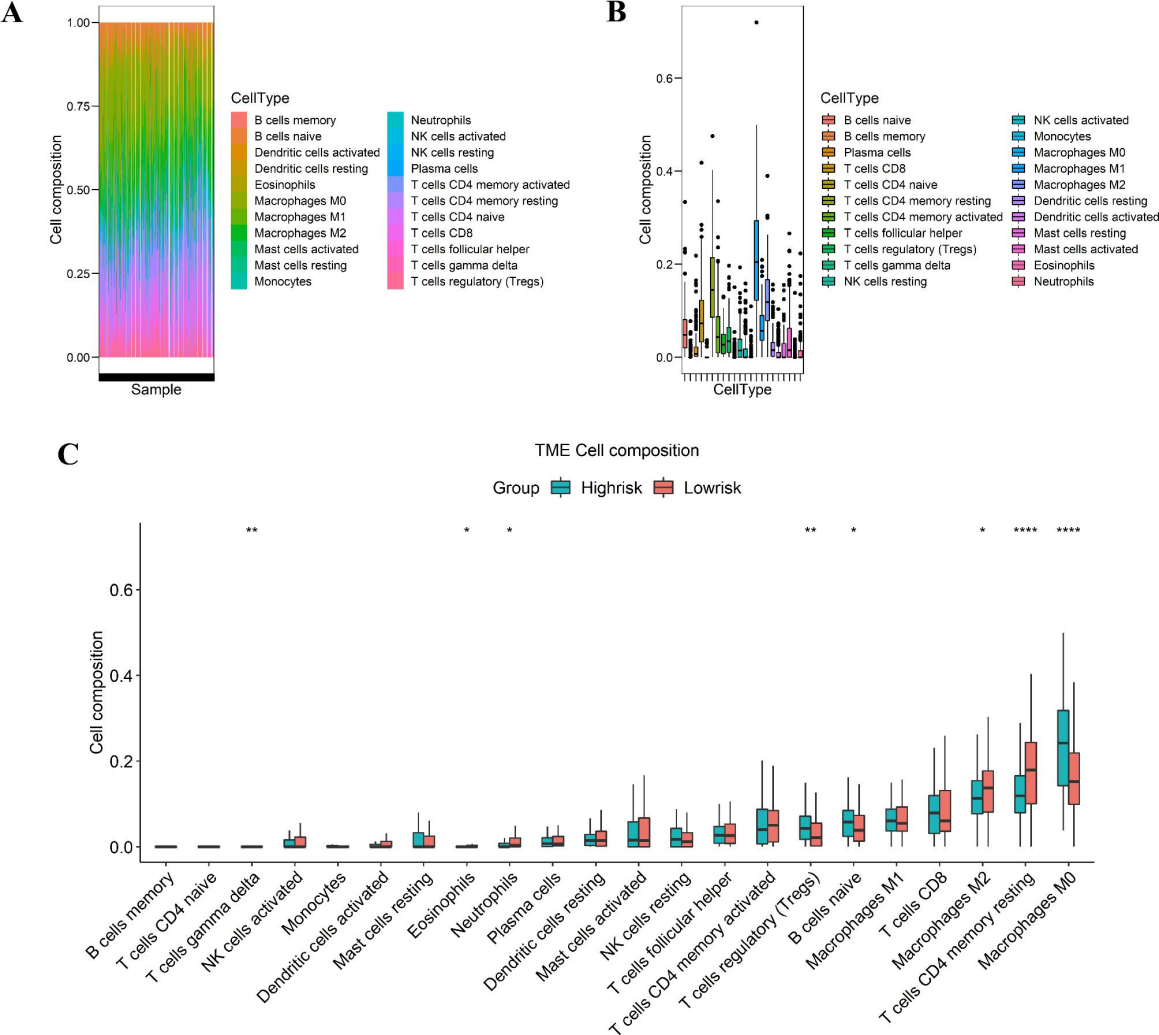
Immune cell subtypes in CRC were analyzed using CIBERSORT. (A) Bar chart displaying the proportion of immune cell subsets. The x-axis shows sample names, and the y-axis shows the percentage of 22 immune cell subsets; (B) Box plot of the percentage of 22 immune cell subsets; (C) Proportion of 22 immune cell subsets in the high- and low-risk groups. ^***^p < 0.001, ^**^p < 0.01, ^*^p < 0.05

Increasing evidence shows that the prognosis and clinical outcome of CRC patients are strongly influenced by immune cell infiltration in the tumor microenvironment [16]. Comprehensive evaluation of immune infiltrates integrating both the quantity and variety of tumor-infiltrating immune cells and incorporation of composite scores encompassing clinically biomarkers, is emerging as a promising strategy potentially capable of risk stratification and optimizing patient selection [17]. Thus, to further investigate the correlation between immune cell infiltration and the prognostic signature, we used the CIBERSORT algorithm to calculate the content of 22 immune cell populations in each CRC sample by setting p < 0.05 as the threshold for screening. The results showed that macrophages M0 were the most abundant infiltrating cells, followed by CD4 memory T cells (Fig. 7 A, B). We also observed differences in immune cell infiltration between the two risk groups. As the results showed in Fig. 7 C, the proportion of T cells gamma delta, eosinophils, neutrophils, macrophages M2, and CD4 memory T cells were significantly enriched in the low-risk group compared to the high-risk group (p < 0.05), while a high proportion of regulatory T cells (Tregs), naïve B cells, and M0 macrophages were significantly in the high-risk group than in the low-risk group (p < 0.05).

### Experimental verification of key genes by qRT-PCR and IHC

**Fig. 8:**
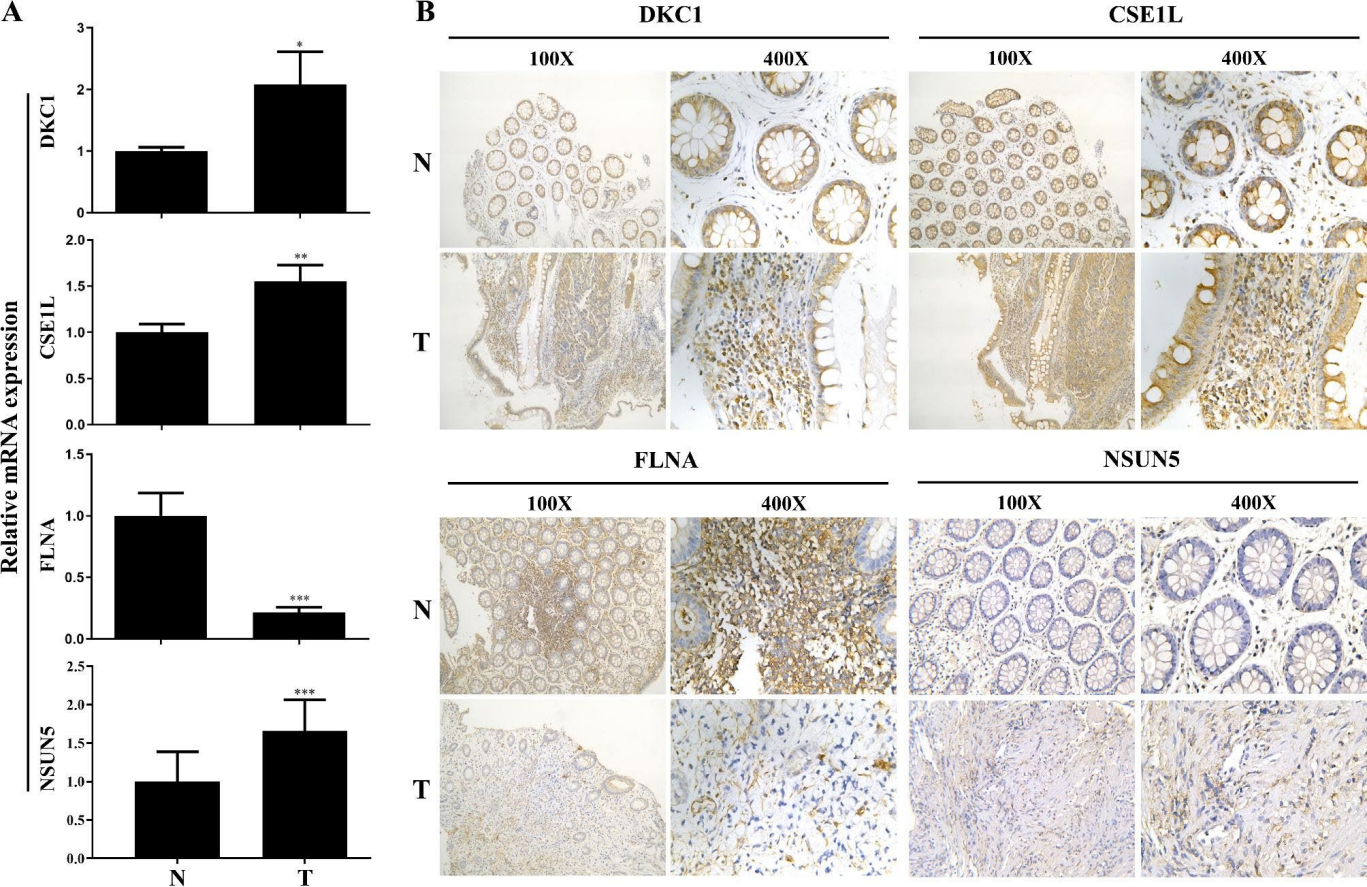
The mRNA and protein expression of the four hub genes in CRC tissues and normal tissues. (A) The mRNA expression of DKC1, CSE1L, FLAN and NSUN5 were analyzed by RT-PCR, ^***^p < 0.001, ^**^p < 0.01, ^*^p < 0.05; (B) Protein levels of the four genes in CRC tissues and normal tissues were analyzed by IHC assay. N: normal tissues, T: CRC tissues

In order to further analyze the expression of the hub genes in patients, CRC and adjacent normal tissues of patients were collected (Supplemental material Table S2). To analyze the mRNA levels of DKC1, FLNA, CSE1L and NSUN5, qRT-PCR was performed. The mRNA levels of DKC1, CSE1L and NSUN5 were higher in the colon cancer tissues than in the paired adjacent normal tissues, while the level of FLNA was lower (p < 0.05, Fig. 8 A). IHC staining of the five pairs of CRC and adjacent normal tissues showed that DKC1, CSE1L and NSUN5 expression levels were significantly higher in the CRC tissues compared to the paired adjacent normal tissue, while FLNA protein level was significantly lower in the CRC tissues (Fig. 8B). Taken together, these results suggested that DKC1, CSE1L and NSUN5 were dramatically overexpressed while FLNA was significantly downregulated in colorectal cancer.

## Discussion

CRC is the most common cancer worldwide, with the third-highest mortality rate [18]. It has been one of the leading causes of cancer-related deaths, and hence, the prognosis of CRC has always been a major concern. Over the past few decades, although the traditional therapies and combination chemotherapy has had a significant effect on the CRC, the treatment of recurrent and metastatic CRC patients did not improve [19–21]. Meanwhile, there is a lack of research on the molecular mechanism associated with metastasis of CRC [22–24]. Therefore, developing new biomarkers with high prognostic values to estimate the treatment response and survival outcomes of CRC patients is crucial. In this study, bioinformatics was used to screen the CRC-related genes based on the gene expression data of CRC patients in the TCGA database. A total of 6819 DEGs were explored from the TCGA database, followed by MEGENA and PPI to deduce the gene co-expression networks. A total of 337 candidate genes were identified. Finally, based on the survival analysis of the 337 candidate genes, we screened four-gene expression signatures, including DKC1, NSUN5, FLNA and CSE1L.

Diagnosis and prediction are the most important steps in the management of CRC patients. To screen the candidate biomarkers that may be helpful for the diagnosis and prognosis for CRC, a four-gene signature was identified by our data processing system. Then, these genes were used to construct a diagnostic model. The AUCs of the four-gene signature showed a perfect diagnostic ability in CRC gene expression samples in the training and test sets from the TCGA database. Next, to provide a robust indicator for the prognostic evaluation on the DFS and OS with CRC, we also constructed a prognostic model and illustrated the impact of the prognostic signature for CRC patients, based on a combination of the risk score distribution, survival status scatter plot, and gene expression heatmap. In addition, the expressions of DKC1, NSUN5, FLNA and CSE1L were validated by qRT-PCR and IHC, and found that DKC1, CSE1L and NSUN5 were highly expressed and FLNA was underexpressed in CRC tumor tissues, consistent with the data in TCGA database. These results suggested that expression changes of DKC1, NSUN5, FLNA and CSE1L could be associated with the the prognosis of CRC patients. Because the number of samples collected in this study is limited, it can be used as a basis for prognosis and diagnosis of the hub genes in CRC patiens. Large clinical specimens to further evaluate the value of the four hub genes as a prognostic and diagnostic evaluation indicator of CRC is our next major study objectives.

DKC1 is an X-linked gene which encodes dyskeratosis protein on the X chromosome (Xq28) and participates in the occurrence of congenital keratosis [25]. DKC1 is a major component of telomerase ribonucleoprotein complex and has a major impact on the functional stability of telomerase ribonucleoprotein complex. The upregulation of DKC1 is closely related to poor prognosis in prostate cancer, neuroblastoma, and hepatocellular carcinoma [26–28]. FLNA is located on the X chromosome and is a critical signal transduction scaffold protein [29, 30]. Previous studies have shown that FLNA promotes cell proliferation, migration, and invasion [31]. However, some studies have reported that FLNA is significantly upregulated in cervical cancer, and has a good predictive effect, which can be used as a prognostic signature of cervical cancer [32]. The involvement of chromosome 20 in human cancers is well-documented. CSE1L is mapped to 20q13, which encodes a protein that mediates the nuclear export of importin-α [33, 34]. CSE1L is differentially expressed in various malignant tumors and is related to the ability of tumor invasion, metastasis, and proliferation [35–37]. NSUN5 is generally upregulated in human cancers, including CRC, which may be attributed to the hypomethylation of NSUN5 promoter. The cancer patients with an overexpressed NSUN5 have a poorer prognosis, and this condition is positively correlated with NSUN5 translation. Epidemiologic study demonstrated that high expression of NSUN5 was associated with advanced tumor stages (III, IV), which possibly relate to its promotion of cell proliferation through regulating cell cycle in CRC [38]. Our study provides a different approach to establishing the prediction model and selecting the candidate oncogenes or biomarkers that exhibit a marked capacity for diagnosis and prognosis of CRC.

In order to elucidate the mechanisms underlying the signature, we performed GSEA to analyze the KEGG pathways between the two risk groups. The most significantly enriched pathways in the high-risk group were focal adhesion, ECM receptor interaction, AXON guidance, basal cell carcinoma, and WNT signaling pathway. These pathways were considered to be associated with the poor prognosis of CRC [11–15]. These results suggested that the interaction of these pathways and the four hub genes (DKC1, NSUN5, FLNA and CSE1L) perhaps an important reason for the poor prognosis in the high-risk group.

Furthermore, the clinical features of CRC are vital factors that influence the prognosis of patients. In this study, we established a robust prognostic signature, which could categorize CRC patients into high- and low-risk groups with statistically different OS outcomes. Next, we systematically analyzed the patient risk score and clinical information in TCGA (including age, gender, tumor stage, T stage, and N stage) for the OS by univariate and multivariate Cox regression analysis. The results indicated that our signature could be used as an independent prognostic factor to predict the prognosis of patients. In terms of clinical relevance, the prognostic signature was significantly correlated with gender.

Additionally, epidemiologic studies had revealed that the immune microenvironment affect the development and prognosis of colorectal cancer [39]. Zhang et al. found that the patient with a higher degree of tumor immune invasion had a better clinical prognosis [40]. Therefore, exploring the correlation between prognosis and tumor immune function has guiding significance for the diagnosis and treatment of colorectal cancer. The current signature identified an association between the increased number of M0 macrophages in the high-risk patients and CD4 memory resting T cells, which was significantly higher in the low-risk group than in the high-risk group. We also observed differences in immune cell infiltration between the two risk groups. However, additional data are required to draw a conclusion. Previous studies focused on macrophages and Tregs in the immunological landscape of CRC. Tregs may contribute to cancer development, and macrophages may be associated with cancer metastasis and immune suppression. Zhang et al. found that CD4 memory resting T cells were associated with patients with advanced CRC, indicating that these cells predicted survival [41]. Jiang et al. indicated that patients with high numbers of M0 macrophages in the tumor environment have an increased risk of mortality [42]. A possible explanation is that M0 macrophages, together with other suppressor cells, such as Tregs, contribute to an immunosuppressive environment. Taken together, the infiltration of immune cells in CRC indicates the immune status of patients, which might underlie the difference in survival outcomes between the two risk groups.

Nevertheless, the present study has some limitations. Firstly, our findings are based on public databases with a limited number of patients. Secondly, this is a retrospective study, and prospective studies are needed to verify our signature. Finally, we initially used a small number of clinical samples to verify the genes screened by the model. However, more clinical samples, basic experiments and the molecular mechanisms for this signature need to be further substantiated in future studies.

## Conclusion

In conclusion, we constructed a diagnostic model based on the SVM. Our results revealed that this model has a relatively high diagnostic efficiency for CRC. Moreover, we identified a 4-gene prognostic signature as potential prognostic predictor for CRC patients. These findings would provide a theoretical reference for the future exploring the potential biomarkers for diagnosis and prognosis prediction of CRC patients.

## Materials and methods

### Collection of clinical samples

Five human CRC and adjacent normal tissues were obtained from patients diagnosed with CRC and received surgery at the People’s Hospital of Longhua, Shenzhen, from October to November 2021. No patient had received preoperative chemotherapy or radiotherapy. The procedure of this study was approved by the Ethics Committee of the People’s Hospital of Longhua, Shenzhen (approval number: Ethical review of the People’s Hospital of Longhua (Institute) [2021] No. 120), and the utilization of clinical samples followed the guidelines of the Ethics Committee of the hospital. Informed consent was obtained from all participants.

### Data download

We downloaded CRC RNA-seq raw count data from the TCGA database (https://portal.gdc.cancer.gov), which were preprocessed and normalized using the R package “Deseq2” and R package “pre-process Core.” The clinical information of CRC patients was downloaded from cBioPortal (http://www.cbioportal.org/).

### Data preprocessing and DEGs analysis

Differential analysis between CRC tissues and non-cancerous adjacent tissues was performed using the R package “limma.” Then, the Benjamini–Hochberg method was utilized for differential analysis to obtain DEGs. Fold-change (FC) and adjusted p-values (adj. p-value) were used to screen the DEGs. |log (FC)| ≥ 1 and adj. p-value < 0.05 were defined as the screening criteria for DEGs [43].

### MEGENA of DEGs

MEGENA is an algorithm for mining module information from expression spectrum data [44]. According to the algorithm, modules are defined as a group of genes with similar expression profiles. If some genes always have similar expression changes in a physiological process or different tissues, they are defined as a module. MEGENA consists of three major steps: (1) The correlation between any two genes is calculated, and the genes are sorted according to the correlation; (2) Fast Planar Filtered Network construction (FPFNC) by introducing parallelization, early termination, and prior quality control; (3) PFNs are iteratively processed by three criteria: the shortest path distance, local path index, and overall modularity to obtain accurate gene classification.

### PPI analysis of node genes

The node genes were obtained by MEGENA algorithm. The PPI analysis of the node genes was performed using String10.0. The results of the analysis were established using a gene expression network and mapped by R package “igraph” and R package “ggraph”. The interaction correlation (degree ≥ 10) was taken as the criterion to screen out the hub genes with high connectivity in the gene expression network.

### Survival analysis of hub genes

The R package survival was applied to explore the roles of hub genes in disease-free survival (DFS) based on the tumor sample from the TCGA database. CRC patients were divided into two groups based on the median expression values of the hub genes. Then, the Kaplan–Meier (K–M) survival curve was established by combining the survival information of CRC patients in the high- and low-expression groups. p < 0.01 was considered statistically significant by the log‑rank test.

### Construction and validation of the diagnostic model by SVM

The significant risk genes were screened out through survival analysis. An SVM model was trained using ten-fold cross-validation [45]. The SVM model is a supervised classification algorithm of machine learning using python (version 3.8) package scikit-learn, which distinguishes and predicts the samples through Eigenvalues of the candidate genes in each sample and evaluates the probability of belonging to a specific category, thereby realizing the prediction between CRC tumor and normal samples. The true and false-positive rates were estimated, and the area under the ROC curve (AUC) was employed to estimate the performance of the model on the training and test sets from the TCGA database.

### Establishment and validation of the prognostic model

Patients with clinical information (age, gender, stage, N stage, T stage, and OS time) were contained in the subsequent prognostic analysis. A total of 589 patients from the TCGA database were analyzed for the clinical correlation. These patients were divided into the training and test sets at a ratio of 1:1 using the R package “caret.” The training set was used to identify the survival-related DEGs and establish a prognostic signature, while the test and the total sets were used as internal validation sets.

The formula of the risk score for the prediction of CRC patients’ prognosis was as follows: risk score = ∑ β*i* * expgenei (β*i*: the coefficient of expgene*i*. expgene*i*: the expression level of gene*i*). CRC patients were divided into high-risk and low-risk groups based on the median risk score of the training set. The survival analysis of the two groups was based on DFS and OS. The K–M survival curves were drawn using the R package “survival” and “survminer.” Next, we calculated the values of AUC to verify the feasibility and accuracy of our prognostic model and the clinical characteristics in predicting the patients’ prognosis. Finally, both univariate and multivariate Cox regression analyses were conducted using clinical parameters and risk scores to evaluate the independent prognostic value of the signature.

### Gene set enrichment analysis

Patient samples were divided into high- and low-risk groups based on the risk score. Then, GSEA were performed to identify the KEGG pathways in the two groups using the Molecular Signatures Database (http://www.gsea-msigdb.org/gsea/downloads.jsp). At p < 0.05, normalized enrichment scores (NES) > 1.0, and a false-discovery rate (FDR) q < 0.25, the pathway was considered significant.

### Estimation of infiltrating immune cells

Tumor-infiltrating immune cells (TIICs) were estimated between the two risk groups using the CIBERSORT algorithm [46]. The algorithm utilized normalized gene expression data and the annotated gene signature matrix (LM22) to determine 22 immune cell subtypes. The LM22 file was downloaded from the CIBERSORT web portal (https://cibersort.stanford.edu/). Samples from CIBERSORT (p < 0.05) were considered significant and screened for further analysis.

### Quantitative real-time polymerase chain reaction (qRT-PCR) analysis

Total RNA was extracted from clinical tissue samples (cancer and pared normal tissue) using TRIzol reagent (Invitrogen, Carlsbad, CA, USA). An equivalent of 1 µg of RNA was reverse transcribed into cDNA with oligo (dT) primers using the cDNA synthesis kit (Takara, Dalian, China). qRT-PCR was performed using SYBR Green qPCR Master Mix (Roche, Shanghai, China) according to manufacturer’s instructions. *β*-actin was used as a housekeeping gene for normalization, and the sequences of the qRT–PCR primers are listed in Supplementary material Table S3. The qRT-PCR amplification reaction was as follows: initial denaturation at 95 °C for 10 min, followed by 40 cycles of 95 °C for 15 s and 60 °C for 45 s. The reactions were carried out in triplicates, and the relative quantification was performed using the comparative CT (2^−∆∆CT^) method.

### Immunohistochemistry (IHC) staining analysis

Tumor and paired normal tissue from the CRC patients were fixed in 10% formalin. Following standard protocols, 3-µm-thick sections were prepared and stained. The IHC staining was conducted using the UJltraSensitive™ SP (Mouse/Rabbit) IHC kit (MX Biotechnologies, Fuzhou, China), which contained endogenous peroxidase blocking solution, serum, secondary antibody, streptavidin-peroxidase, and DAB substrate-chromogen. The tissue sections were incubated with the rabbit polyclonal antibodies of anti-DKC1 (1:100, Servicebio, Wuhan, China), anti-FLNA (1:100, Abcam, MA, USA), anti-NSUN5 (1:200, Abcam, MA, USA) and anti-CSE1L (1:100, Abcam, MA, USA) overnight at 4 °C.

### Data analysis and statistics

Statistical analyses of this study were conducted using the R software. The ROC curve analysis with the AUC was utilized to assess the predictive performance of the diagnostic and prognostic model. K–M curves with the log-rank test were plotted using the R package survival program. Additionally, univariate and multivariate Cox regression analyses were utilized to confirm the independent prognostic factors within clinicopathological characteristics. P < 0.05 indicated statistical significance for all analyses [47].

## Electronic supplementary material

Below is the link to the electronic supplementary material.

Supplementary Material 1: Table S1: Characteristics of CRC patients included the prognostic model of DFS and OS in TCGA cohort

## Data Availability

The original contributions presented in the study are included in the article. Further inquiries can be directed to the corresponding authors.
