# Supplementary material for: Identification and validation of a novel signature as a diagnostic and prognostic biomarker in colorectal cancer
Source: Biol Direct. 2022 Nov 2;17:29. doi: 10.1186/s13062-022-00342-w (PMC9628086; doi:10.1186/s13062-022-00342-w)
Supplement: Supplementary file 1 — Supplementary Material 1: Table S1: Characteristics of CRC patients included the prognostic model of DFS and OS in TCGA cohort [file 13062_2022_342_MOESM1_ESM.docx]

**Supplementary material:**

**Table S1 Characteristics of CRC patients included the prognostic model of DFS and OS in TCGA cohort**

| Variables | | The prognostic model of OS | | | | | | | | | The prognostic model of DFS | | | | | | |
| --- | --- | --- | --- | --- | --- | --- | --- | --- | --- | --- | --- | --- | --- | --- | --- | --- | --- |
|  |  | Training cohort | | | Testing  cohort | | TCGA  cohort | | | Training  cohort | | Testing  cohort | | | | TCGA  cohort | |
| Riskscore |  | | |  | | | | |  |  | | |  | | | |  |
| Low risk | 146 | | 125 | | | 271 | | | | 55 | | | 44 | | | | 99 |
| High risk | 147 | | 168 | | | 315 | | | | 56 | | | 66 | | | | 122 |
| Age |  | | |  | | | | |  |  | | |  | | | |  |
| <60 | 61 | | 63 | | | 124 | | | | 30 | | |  | 43 |  | | 73 |
| ≥60 | 232 | | 230 | | | 462 | | | | 81 | | |  | 67 |  | | 148 |
| Stage |  | | |  | | | | |  |  | | |  | | | |  |
| I | 53 | | 57 | | | 110 | | | | 19 | | |  | 25 |  | | 44 |
| II | 110 | | 106 | | | 216 | | | | 45 | | |  | 51 |  | | 96 |
| III | 80 | | 79 | | | 159 | | | | 29 | | |  | 29 |  | | 58 |
| IV | 50 | | | 51 | | | | 101 | | 18 | | | 5 | | | | 23 |
| N stage |  | | |  | | | | |  |  | | |  | | | |  |
| N0 | 167 | | 175 | | | 342 | | | | 69 | | | 80 | | | | 149 |
| N1 | 64 | | 59 | | | 123 | | | | 22 | | | 22 | | | | 44 |
| N2 | 62 | | 59 | | | 121 | | | | 20 | | | 8 | | | | 28 |
| T stage |  | | |  | | | | |  |  | | |  | | | |  |
| T1 | 10 | | 10 | | | 20 | | | | 2 | | | 6 | | | | 8 |
| T2 | 46 | | 59 | | | 105 | | | | 20 | | | 26 | | | | 46 |
| T3 | 200 | | 193 | | | 393 | | | | 75 | | | 70 | | | | 145 |
| T4 | 37 | | 31 | | | 68 | | | | 14 | | | 8 | | | | 22 |
| gender |  | | |  | | | | |  |  | | |  | | | |  |
| Female | 148 | | 131 | | | 279 | | | | 48 | | | 54 | | | | 102 |
| Male | 145 | | 162 | | | 307 | | | | 63 | | | 56 | | | | 119 |

**Table S2 Clinical data of the five patient donors**

| **No.** | **Patient ID** | **Gender** | **Age (years)** | **Diagnosis** | **Histologic grade** | **TNM stage** | **Site** |
| --- | --- | --- | --- | --- | --- | --- | --- |
| **1** | 5067236 | Male | 48 | CRC | Poorly differentiated adenocarcinoma | T3N1cM0 | rectum |
| **2** | 6380278 | Female | 64 | CRC | Moderately differentiated adenocarcinoma | T1bN0M0 | sigmoid |
| **3** | 3073056 | Female | 48 | CRC | Moderately differentiated adenocarcinoma | pT3N0M0 | rectum |
| **4** | 5476572 | Female | 43 | CRC | Moderately differentiated adenocarcinoma | T2N0M0 | rectum |
| **5** | 2098577 | Female | 74 | CRC | Undifferentiated adenocarcinoma | T3N0M0 | rectum |

| **Genes** | **Primer sequences** |
| --- | --- |
| *β*-actin | F：TGGCACCCAGCACAATGAA |
|  | R：CTAAGTCATAGTCCGCCTAGAAGCA |
| DKC1 | F：GAGTTACCTGCGGCGAGTTG |
|  | R：TTGACCTCAATGCCGTCCTC |
| FLNA | F：ACATCATCGACCACCATGACA |
|  | R：CTCCTGGTCTTTGCCAACGTC |
| CSEIL | F：TCCCCAATGATGACACTGA |
|  | R：TCCCAGAAATGCAACTGT |
| NSUN5 | F：CTCGTCTACTCCACGTGCTC |
|  | R：TCGACCCGTTCAATTACAGCA |

**Table S3 The qRT–PCR primer sequences used in the study**
